# High-Barrier, Biodegradable Films with Polyvinyl Alcohol/Polylactic Acid + Wax Double Coatings: Influence of Relative Humidity on Transport Properties and Suitability for Modified Atmosphere Packaging Applications

**DOI:** 10.3390/polym15194002

**Published:** 2023-10-05

**Authors:** Antonio Barbato, Annalisa Apicella, Francesca Malvano, Paola Scarfato, Loredana Incarnato

**Affiliations:** Department of Industrial Engineering, University of Salerno, Via Giovanni Paolo II, 132, 84084 Fisciano, Italy; abarbato@unisa.it (A.B.); fmalvano@unisa.it (F.M.); pscarfato@unisa.it (P.S.); lincarnato@unisa.it (L.I.)

**Keywords:** biodegradable film, polylactic acid (PLA), polyvinyl alcohol (PVOH), double coating, O_2_ and water permeability, permselectivity, modified atmosphere packaging, food packaging, modeling, water resistance

## Abstract

Polyvinyl alcohol (PVOH) exhibits outstanding gas-barrier properties, which favor its use as a biodegradable, high-barrier coating on food-packaging films, possibly in combination with modified atmospheres. Nonetheless, its high sensitivity to water can result in a severe loss of barrier properties, significantly limiting its applications with fresh foods and in high-humidity conditions. In this work, the water vapor (P_WV_) and oxygen permeability (PO_2_) of high-barrier biodegradable films with PVOH/PLA + wax double coatings were extensively characterized in a wide range of relative humidity (from 30 to 90%), aimed at understanding the extent of the interaction of water with the wax and the polymer matrices and the impact of this on the permeation process. What is more, a mathematical model was applied to the P_WV_ data set in order to assess its potential to predict the permeability of the multilayer films by varying storage/working relative humidity (RH) conditions. The carbon dioxide permeability (PCO_2_) of the films was further evaluated, and the corresponding permselectivity values were calculated. The study was finally augmented through modified atmosphere packaging (MAP) tests, which were carried out on double-coated films loaded with 0 and 5% wax, and UV-Vis analyses. The results pointed out the efficacy of the PLA + wax coating layer in hampering the permeation of water molecules, thus reducing PVOH swelling, as well as the UV-shielding ability of the multilayer structures. Moreover, the MAP tests underlined the suitability of the double-coated films for being used as a sustainable alternative for the preservation of foods under modified atmospheres.

## 1. Introduction

Plastic pollution is an issue of major concern in our society today. According to the 2022 OECD Global Plastic Outlook [1], about 22% of all plastic waste worldwide ends up in landfills or as litter, causing significant harm to land and marine environments. Biodegradable polymers are a viable alternative to conventional plastics from a sustainability and circular economy perspective [2]; however, their performance needs to be enhanced to meet the needs of the market, especially in those sectors where plastic consumption is very high [3].

Polyvinyl alcohol (PVOH) has gained considerable attention among bioplastics due to its unique qualities, including its rapid biodegradability in suitable environments [4,5,6,7], good tensile strength, biocompatibility, and outstanding gas-barrier properties [8,9,10,11]. These attributes make it highly valuable especially in the food-packaging sector, where providing an adequate gas barrier through active and/or passive technologies is of utmost importance in order to control gas exchange, reduce food oxidation phenomena, and stabilize headspace composition in MAP systems [12,13,14,15,16].

PVOH is generally combined in multilayer structures realized by coating [8,17,18], coextrusion [19,20], and electrospinning [21,22] with the aim of joining gas barrier, heat resistance, printability, and antioxidant properties [23,24,25,26]. The coating technology, in particular, is garnering a wide interest within scientific and industrial research because it allows one to functionalize biodegradable substrate films in a simple and effective way, conferring them an advanced performance without modifying their bulk properties nor their biodegradable and/or recyclability features [27,28].

Despite its excellent gas barrier, the main limitations of PVOH, like other hydrophilic biopolymers, are its poor water vapor barrier properties: indeed, water plasticizes the polymer matrix, significantly affecting the absorption and diffusion of even other gases and weakening the structural integrity. This precludes its use in high-humidity environments or in contact with moist foods [29,30,31].

Therefore, the study and mathematical modeling of the mechanisms governing mass diffusion in PVOH packages at different relative humidities becomes an essential task to predicting their final performance and determining their suitability for preserving the quality of packaged foods under real working conditions [32,33,34]. To the best of our knowledge, only a few studies have described and modeled the effect of relative humidity (RH) on PVOH permeability. Abdullah et al. [35] studied the water- and gas-barrier properties of PVOH/starch/glycerol + halloysite nanotubes (HNT) nanocomposite films. The authors found that the water vapor permeability coefficient increased linearly as the RH gradient increased, following the typical trend of materials that obey Fick’s law. Mo et al. [36] investigated the effects of RH and temperature on the barrier properties of bi-oriented polyvinyl alcohol (BOPVOH) films, finding that the relationship between water vapor and oxygen permeability and RH% was well expressed by an exponential trend. 

In our previously published work [8], biodegradable films with a high gas-barrier performance were realized by spreading a double coating layer of modified PVOH (m-PVOH) and PLA + ethylene-bis-stereamide (EBS) wax (from 0 to 20%) on a poly(butylene-adipate co-terephthalate) (PBAT)/poly(lactide) (PLA) substrate. The films combined the ductility of the PBAT/PLA web layer with the high oxygen barrier of the m-PVOH and the heat sealability and water-resistance of the PLA + EBS wax layer, with a potentially positive effect on the water vapor barrier properties.

In this work, our goal was to investigate how ambient humidity affects the transport properties of these multilayer structures, and the impact of the films’ configuration on the final barrier performance.

The water vapor and oxygen barrier properties were determined at ambient temperature (23 °C) over a wide range of relative humidities. Additionally, a mathematical model was applied to the data set to describe the trends of P_WV_ evolution by increasing the RH and assess its potential for predicting the barrier performance of the multilayer systems at different humidities. The carbon dioxide permeabilities were determined and the suitability of the developed films for MAP were assessed. The optical properties of the films were finally measured to evaluate the UV-shielding ability, which plays a fundamental role in preserving sensitive foods from light-induced deterioration mechanisms.

## 2. Materials and Methods

### 2.1. Materials

A blown web layer made of a commercial PBAT/PLA blend (Biofilm) with the trade name Bioter^TM^ (Euromaster S.p.a., Pistoia, Italy) was chosen as the substrate.

Exceval AQ-4104 is a polyvinyl alcohol modified with ethylenic insertions that exhibits a low gas barrier from dry conditions to approximately 55% relative humidity (RH%), according to the technical data provided by the manufacturer. It is totally hydrolyzed (98.0–99.0 mol%), chlorine-free, and water-soluble, and it has received FDA approval for food-contact use. The polymer will be hereafter named m-PVOH.

PLA4060D (amorphous, D-isomer concentration = 12 wt%, Mw 190,000 g/mol, specific gravity = 1.24 g/cm^3^) was provided by Natureworks (Minnetonka, MN, USA).

Deurex (Elsterau, Germany) supplied the Ethylene-bis-Stearamide (EBS) wax X2010M (CAS 110–30-5), produced by revaluing sugar cane residues. The wax is approved for the production of commodities intended to come into contact with food, according to the EU Regulation 10/2011 and the FDA 21 CFR [37], and is classified as a no-hazard substance by the European Chemicals Agency (ECHA) [38]. Analytical-grade solvents were utilized throughout.

### 2.2. Production of the Double-Coated Films

The double-coated films were realized with the same methodology and conditions reported by Apicella et al. [8]. Briefly, the Biofilm substrate monolayer, having a thickness of 22 ± 2 μm, was realized using a GIMAC lab scale blown film plant, outfitted with a single-screw extruder (D = 12 mm, L/D = 24) with a thermal profile set between 190 °C and 160 °C, a screw speed of 50 rpm, and a collection speed of 3 m/min. The substrate was then double coated using a laboratory bar coater (RK, Printocoat Instruments Ltd., Litlington, UK), equipped with a stainless steel closed-wound rod with a wire diameter equal to 0.80 mm. Firstly, the m-PVOH/water solution (mass ratio 10:90) was prepared, spread on the web, and dried at 120 °C for 4 min in an oven. Then, the second layer made using a PLA/acetone solution (20:80) loaded with 0%, 5%, 10%, and 20% w/w_PLA_ EBS wax was deposited and dried at room temperature. In the present study, unlike the previously published work by Apicella et al. [8], the thickness of the m-PVOH layer was increased from 5 ± 1 μm to 10 ± 1 μm to further improve the barrier performance of the films. Table 1 reports the list of the realized films and the relative thicknesses of the layers.

### 2.3. Water Vapor Permeability and Modeling

The water vapor transmission rate (WVTR) measurements of the films were performed through a Water Vapor Permeation Analyzer (Model 7002—Systech Illinois, Princeton, NJ, USA) at 23 °C and at 30%, 50%, 70%, 85%, and 90% relative humidity. All the measurements were carried out in triple according to the ASTM F 1249-90 standard. The area of the tested films was 5 cm^2^. The values of the water vapor permeability coefficients (P_WV_) were calculated assuming that the double-coated films were homogeneous [39] and applying the following equation:

(1) $$P_{WV}=\frac{WVTR\times l}{\Delta P}$$

where *WVTR* is the water vapor transmission rate (g/m^2^ day) of the film, l is the average film thickness (mm), and ∆P is the partial water vapor pressure difference (bar) at the two sides of the film.

In order to describe and predict the P_WV_ behavior as a function of relative humidity for the developed systems, the P_WV_ data were fitted using an exponential mathematical model, using the software CurveExpert Professional^©^ 2.7 to solve the equation. The fit was optimized based on the minimization of the sum of the squared residuals, and the coefficients of the determination R^2^ were calculated as a measure of the goodness of the fit.

### 2.4. Gas Permeability Measurements and Permselectivity

The oxygen transmission rate (OTR) and carbon dioxide transmission rate (CO_2_TR) tests were conducted by means of a gas permeabilimeter (GTT, Brugger, Munich, Germany) following the ISO 15105-1 standard. The measurements were carried out in triple on film specimens with an area of 16 cm^2^; the gases’ flow rate was set at 80 mL/min.

The OTRs were evaluated at 23 °C and at three different levels of RH% (10%, 50%, and 85%) by conditioning the oxygen insufflated in the measuring chamber through paper filters soaked with different saturated salt solutions of known water activity (a_w_), while the CO_2_TR measurements were performed at 23 °C and 50% RH. 

The oxygen permeability coefficients (PO_2_) and carbon dioxide permeability coefficients (PCO_2_) were calculated by multiplying the measured OTRs and CO_2_TRs, respectively, by the thickness in mm of the samples, assuming that the double-coated films were homogeneous [39]. 

The CO_2_/O_2_ permselectivity of the film samples was assessed according to the following formula:

(2) $$Permselectivity=\frac{P_{CO_{2}}\left(23{}^{\circ}C,\;50\%RH\right)}{P_{O_{2}}\left(23{}^{\circ}C,\;50\%RH\right)}$$

### 2.5. Analysis of Packaging Headspace Gas Composition after MAP

Sample bags having 15 × 10 cm size of Bioter/m-PVOH/PLA and Bioter/m-PVOH/PLA + 5%wax were utilized to assess the suitability of the developed films for modified atmosphere packaging (MAP) applications. The bags were filled by gas flushing with a gas mixture consisting of 5% oxygen (O_2_), 30% carbon dioxide (CO_2_), and 65% nitrogen (N_2_), after which they were hermetically sealed. The changes in gas composition within the headspace of the packages were monitored at specific time intervals (0, 7, and 14 days) using an O_2_/CO_2_ gas analyzer (PBI Dansensor, Checkmate3, Ringsted, Denmark). The results were expressed as the average gas concentration (%) ± standard deviation (SD), based on ten replicated bags at each sampling time.

### 2.6. UV-Vis Spectroscopy

The ultraviolet-visible (UV-Vis) spectroscopic measurements were performed on the film samples using a Lambda 800 UV-VIS spectrophotometer (Perkin Elmer, Waltham, MA, USA) in accordance with the ASTM D1746. The transparency of the films was determined by measuring the transmittance at 560 nm. Three replicates of each film were tested. The percent transparency ($T_{R}\%$) was calculated as follows:

(3) $$T_{R}\%=\frac{T_{r}}{T_{0}}\times 100$$

where $T_{r}$ is the transmittance with the specimen in the beam and $T_{0}$ is the transmittance with no specimen in the beam.

The UV-barrier properties were quantified using the average transmittance of UVA (315–400 nm) and UVB (280–315 nm) calculated according to the following equations [40]:

(4) $$UVA\;blocking\;\%=100-\frac{\int_{315}^{400}T\left(\lambda \right)d\lambda }{\int_{315}^{400}d\lambda }$$

(5) $$UVB\;blocking\;\%=100-\frac{\int_{280}^{315}T\left(\lambda \right)d\lambda }{\int_{280}^{315}d\lambda }$$

where *T*(λ) is the average transmittance of the film at the wavelength λ, and *d*λ is the film’s bandwidth.

## 3. Results

### 3.1. Evaluation and Modeling of the Effect of RH on the Water Vapor Barrier Properties of Coated Films

**Figure 1:**
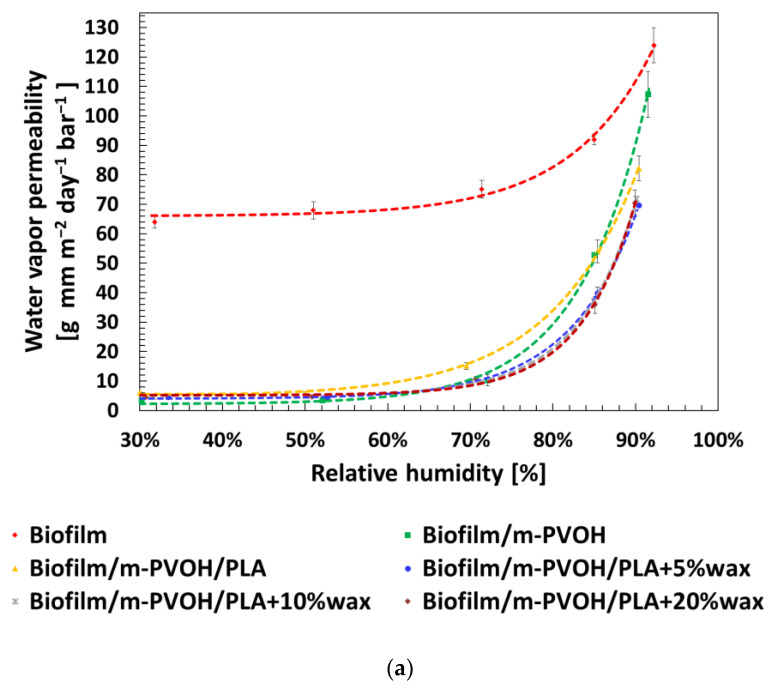

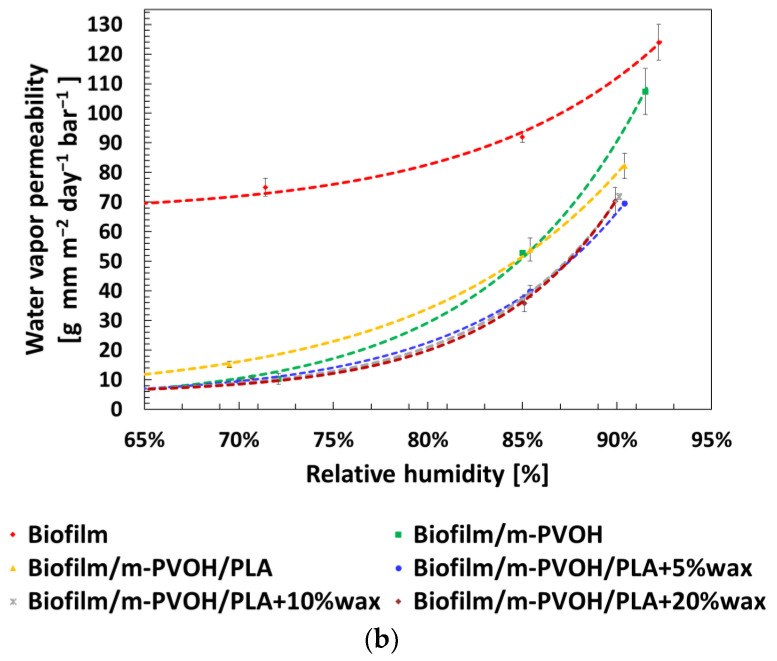
(**a**) Water permeability values at different RH% levels (30%, 50%, 70%, 85%, and 90%) for neat Biofilm and the produced double-coated films. The dotted lines represent the fitting curves. (**b**) Magnification in the range 65–95% RH.

Figure 1a displays the values of P_WV_ for the neat substrate and all the coated films plotted as function of the relative humidity, while Figure 1b provides the magnification between 65 and 95% RH. For a better comprehension, the numerical values of P_WV_ at different RH% are also reported in Table 2.

As observable in Figure 1 and Table 2, for all the tested films, the water vapor permeabilities remained fairly constant until 50% RH and, after, they increase following an exponential growth curve, with some noticeable differences for films with different compositions. These trends can be interpreted by taking into account the fact that the increase in water vapor partial pressure leads to two opposite effects on the transport phenomena through the film: (i) a compaction effect on the polymer chains due to hydrostatic pressure, resulting in an increase in polymer density, which inhibits the diffusion process; and (ii) a plasticization effect as a direct consequence of the increased concentration, which enhances the segmental motions within the polymer and promotes the diffusion [41,42,43]. This is a generally observed behavior in which these two effects are competing; the prevalence of one phenomenon over the other depends on the polymer-penetrant affinity as well as on the relative humidity, and determines the overall permeability of the film to moisture and other molecules. In particular, the Biofilm showed the highest P_WV_ values in the whole RH interval investigated, ranging from 64.0 g mm/m^2^ d bar at 30% RH to 124.0 g mm/m^2^ d bar at 90% RH. These values categorize the substrate as a poor barrier-grade material, according to the classification proposed by Wang et al. [44], and are in line with the literature data reported for films based on PBAT/PLA blends [45,46]. The further spreading of the m-PVOH layer was able to decrease the P_WV_ of one order of magnitude with respect to the neat Biofilm: between 30 and 50% RH, the P_WV_ of Biofilm/m-PVOH film was constant and equal to 3.5 g mm/m^2^ d bar, among the range of high-barrier biodegradable polymers (P_WV_ < 40 g mm/m^2^ d bar) according to Wang et al. [44]. The highwater vapor barrier performance of the m-PVOH coating was attributable to the insertion of ethylenic moieties on the polymer backbone, which were also responsible for the constant barrier properties up to 60% RH, as also reported in the producer’s technical information [8]. Above this critical value, the plasticization of the macromolecular chains prevailed, entailing an increase in the polymer-free volume, a faster diffusion, and, therefore, a steep increase in the P_WV_: indeed, the P_WV_ rose at 10.3 g mm/m^2^ d bar and 107.3 g mm/m^2^ d bar at 70% and 90% RH, respectively. This outcome holds significant relevance in relation to other unmodified PVOH grades, for which the absence of water vapor resistance has been reported even under dry conditions [47], as well as a sharp increase in the P_WV_ (>200%) at a relatively low RH (30–40%) as an effect of the rapid swelling [48,49].

The addition of the amorphous PLA-based second coating layer does not substantially impact the water vapor permeability of the films, since the P_WV_ of the amorphous PLA (~1630 g mm/m^2^ d bar at 23 °C and 50%RH) turned out to be approximately three orders of magnitude larger than that of the Biofilm/m-PVOH support film [50]. Looking at the permeability curves of the Biofilm/m-PVOH/PLA and Biofilm/m-PVOH films, it is interesting to note a crossover point at 85% RH: at this RH, the P_WV_ values of the two films were comparable (equal to 54.0 g mm/m^2^ d bar and 52.8 g mm/m^2^ d bar, respectively), while at 90% RH the Biofilm/m-PVOH/PLA sample exhibited a lower water vapor permeability value (equal to 82.2 g mm/m^2^ d bar) with respect to the Biofilm/m-PVOH (equal to 107.3 g mm/m^2^ d bar). This result can be explained by the fact that in glassy polymers, such as the amorphous PLA, the penetrant molecules occupy specific sites within the polymer’s pre-existing micro voids up to the reaching of a saturation level: at this point, all the available sites are occupied; the permeation phenomenon is no longer dependent on the penetrant-polymer interactions; and the permeation process slows down [51,52].

The inclusion of the EBS wax at different concentrations within the PLA matrix resulted in a reduction in the P_WV_ of the double-coated films with respect to the Biofilm/m-PVOH/PLA sample, as underlined by the ΔP_WV_ % values reported in Table 2. This is attributable to the hydrophobic effect of the EBS wax, which hinders the permeation of water vapor molecules, partially inhibiting the plasticization of the PVOH matrix [53]. The maximum decrease in the P_WV_ ensued after the inclusion of 5% of EBS wax in the PLA matrix and was equal to 37% at 70% RH. Further incorporation of higher percentages of EBS wax into the PLA layer did not substantially change the water vapor barrier’ performance of the double-coated films with respect to the Biofilm/m-PVOH/PLA + 5%wax. These outcomes suggest that, among the developed structures, this latter configuration is the most promising for packaging applications, as it retains the best water vapor barrier performance over such a wide range of relative humidities, with the lowest concentration of wax.

In order to predict the performance of the developed films at specific storage/working RH conditions, it is useful to apply a mathematical model to the data set obtained. In the literature, several authors proposed different models for this purpose. For materials that obey Fick’s law of diffusion, generally, the water vapor permeability increases linearly by increasing the RH. This behavior has been reported for PE and BOPP films [54]. Unlike Fickian materials, the relationship between the RH and the P_WV_ of moderate or high hydrophilic materials, such as most biopolymers, follows an exponential growth curve, according to the following equation [36]:

(6) $$P_{WV}=ae^{b\;RH\%}+c$$

where *a*, *b*, and c are the model parameters. This behavior has been reported for WPI [30], BOPVA [36], alginate, casein, chitosan, and zein films [32]. This latter model was then selected to fit the values: the dotted lines in Figure 1a,b show the best fit of the equation to the P_WV_ data set, while Table 3 gives an overview of the values of the model parameters (a, b, and c) obtained with the fitting procedure, and the R^2^ value as an indicator of the goodness of the fit.

As it can be observed above, the trends of P_WV_ evolution by increasing the RH provided by the model’s numerical solution are in close agreement with the empirical values. In particular, the calculated coefficient of determination R^2^ is higher than 0.994 for all the samples investigated. 

These outcomes underline the perspective to apply this simple mathematical model for predicting the water vapor permeability of the multilayer films over a wide range of relative humidity conditions, and its utility for the characterization, designing, and optimization of their barrier performance depending on the shelf-life requirements of the packaged foods.

### 3.2. Evaluation of the Effect of RH on the Oxygen Barrier Properties of Coated Films

**Figure 2:**
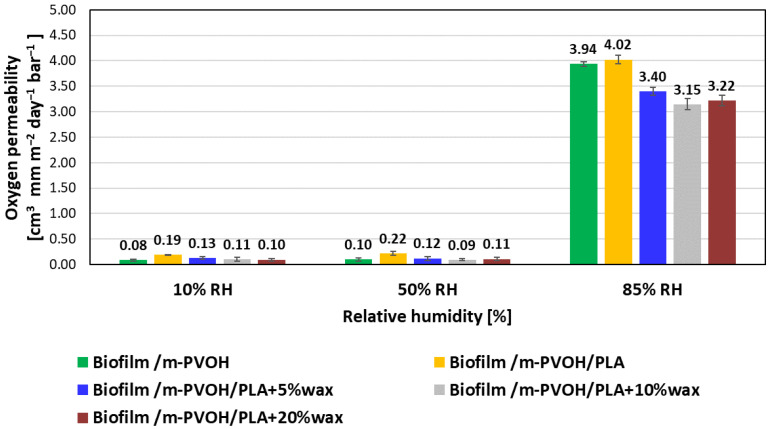
Oxygen permeability (PO_2_) values of all the coated films, evaluated at 10%, 50%, and 85% RH.

The effect of the environmental relative humidity was also assessed on the oxygen barrier of the biodegradable films developed, as oxygen permeation into the package can be responsible for the harmful decay in the safety and quality of packed foods. Figure 2 illustrates the oxygen permeability (PO_2_) values of all the coated films, measured at 10%, 50%, and 85% RH.

Our previously published research [8] highlighted the outstanding oxygen barrier performance obtained after the deposition of a 5 ± 1 μm thick m-PVOH layer, with PO_2_ values comprised between 0.22 and ~0.30 cm^3^ mm/m^2^ d bar for the Biofilm/m-PVOH and the double-coated films, respectively. In the present study, a further improvement in the oxygen barrier performance has been achieved by doubling the thickness of the m-PVOH barrier layer, up to 10 ± 1 μm. In particular, a decrease in PO_2_ values comprised between 42% and 66% for all the coated films was obtained with respect to the results measured by Apicella et al. in 2022 [8].

As already observed for the water vapor permeability coefficients, the oxygen permeabilities remained fairly constant up to 50% RH, thanks to the hydrophobic moieties on the m-PVOH chain structure. At 85% RH, the oxygen permeability values increased by one order of magnitude for all the coated films, compared to the values measured at 10% and 50% RH levels. In a similar fashion to what is reported for the P_WV_ in Figure 1 and Table 2, at 85% RH, the measured PO_2_ for the Biofilm/m-PVOH and Biofilm/m-PVOH/PLA samples were comparable and equal to 3.94 and 4.02 cm_3_ mm/m^2^ day bar, respectively. This outcome confirms that, at this relative humidity, the amorphous PLA coating layer reaches a saturation level which slows down the mass transport through the polymer matrix.

At 85% RH, it is also worth noting that, compared to the pristine Biofilm/m-PVOH/PLA, wax incorporation resulted in a PO_2_ reduction comprised between 15%, for the Biofilm/m-PVOH/PLA + 5%wax, and 22%, for the Biofilm/m-PVOH/PLA + 10%wax. This result suggests that the hydrophobic effect of wax, which reduces the swelling of the m-PVOH layer by restricting the diffusion of water molecules, also allows to partially reduce the permeation of the oxygen molecules.

### 3.3. Carbon Dioxide Barrier Properties, Permselectivity, and Evaluation of Suitability for MAP Application

CO_2_ barrier properties, along with oxygen permeability, are of paramount importance, especially in MAP systems where the levels of both oxygen and carbon dioxide must comply to specific values depending on the food type. CO_2_, when present at moderate concentrations, inhibits enzymatic reactions and delays spoilage, thereby extending the shelf-life of fresh fruits and vegetables [55]. In the case of dry, fatty foods with a low water content (less than 12%), packaging should maintain high levels of carbon dioxide and oxygen levels below 2% in order to effectively inhibit oxidation and prevent rancidity [56]. 

For this reason, in the designing of MAP systems, the permselectivity (PCO_2_/PO_2_ ratio) of the packaging material is a parameter that plays a crucial role, as it must be tailored to create an optimal gas composition within the package, thereby satisfying the specific preservation requirements and extending the shelf-life of packaged food products. 

The CO_2_ permeability and permselectivity values of the Biofilm substrate and all the multilayer films are provided in Table 4. The Biofilm substrate exhibited a remarkably high CO_2_ permeability, equal to 305 cm^3^ mm/m^2^ day bar, which falls between the permeability values of the pristine PLA (~47.5 cm^3^ mm/m^2^ day bar) and PBAT (~390 cm^3^ mm/m^2^ day bar) films reported in the literature [57,58]. The further spreading of the m-PVOH layer reduced the PCO_2_ of the neat substrate by three orders of magnitude: the PCO_2_ of the Biofilm/m-PVOH film came out to be equal to 0.37 cm^3^ mm/m^2^ day bar, highlighting the good barrier properties of m-PVOH also against CO_2_, as reported by other authors [59,60].

Similarly to the water vapor and oxygen permeabilities, the addition of the amorphous PLA had no effect on the barrier against CO_2_, since its PCO_2_ was ~ 47.5 cm^3^ mm/m^2^ day bar, three times larger than the Biofilm/m-PVOH support film [61]. No variations were also appreciable due to the incorporation of the EBS wax, since all the double-coated films exhibited permeability coefficients ranging from 0.44 to 0.46 cm^3^ mm/m^2^ day bar.The calculated permselectivity values for the double-coated films ranged from 2.1, for the Biofilm /m-PVOH/PLA sample, to 4.9, for the Biofilm /m-PVOH/PLA + 10%wax. These values are comparable to those of conventional, fossil-based polymeric films [13,62,63,64,65,66,67] stored under modified atmosphere packaging, with the possibility to tailor the optimal film layout on the basis of the target food’s shelf-life needs.

**Figure 3:**
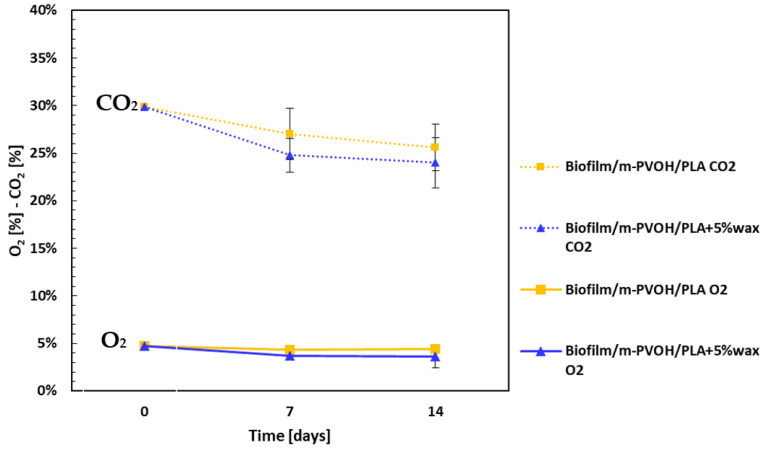
O_2_ and CO_2_ concentrations in the headspace of the Biofilm/m-PVOH/PLA and Biofilm/m-PVOH/PLA + 5%wax bags filled with a 5% O_2_/30% CO_2_/65% N_2_ gas mixture, evaluated at days 0, 7, and 14. Error bars indicate the standard deviation.

To further assess the films’ capability to retain modified atmosphere gas composition over time, MAP tests were carried out on bags made of Biofilm/m-PVOH/PLA and Biofilm/m-PVOH/PLA + 5%wax samples, flushed with a gas mixture consisting of 5% O_2_/30% CO_2_/65% N_2_, hermetically sealed, and stored for up to 14 days. The tests were conducted in the absence of foods that could alter the gas composition due to respiration, solubilization mechanisms in the food matrix, or spoilage effects. The headspace gas composition was monitored during that time and the results, expressed in terms of the measured O_2_ and CO_2_ percentages during that time, are depicted in Figure 3. As it can be observed, the O_2_ concentration remains constant in both the Biofilm/m-PVOH/PLA and Biofilm/m-PVOH/PLA + 5%wax film samples throughout the investigated period. Although the m-PVOH exhibits a good CO_2_ barrier under steady-state conditions, the CO_2_ concentration slightly decreases during that time, from an initial level of 30%, on day 0 to a final value of ca. 25% on day 14, for both samples. However, a slight CO_2_ venting is a desirable condition for the preservation of some foods such as fruits and vegetables in order to avoid an excessive build-up of the gas produced by their respiration up to deleterious levels, which could result in cell membrane damage and physiological injuries to the product [68].

### 3.4. Optical Analysis

**Figure 4:**
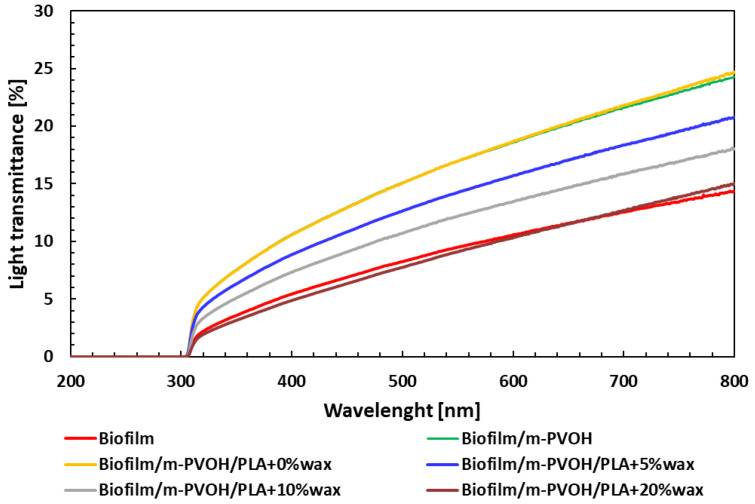
UV-Vis transmittance spectra of all the biodegradable films.

The complete UV-Vis transmittance spectra and T_R_% for the neat Biofilm and all the produced multilayer films are depicted in Figure 4 and Table 5, respectively. As it can be observed, almost zero transmittance was registered in the UVC (200–280 nm) and UVB (280–320 nm) ranges for all the investigated samples, while transmittance remained limited in the UVA (320–400 nm) and visible range, with maximum values around 10–20% at 800 nm, which represents the upper limit of the visible range. The addition of the transparent m-PVOH and PLA layers yielded a slight improvement in the transparency, from 9.6% to 17% and 17.1% for the Biofilm substrate, and the Biofilm/m-PVOH and Biofilm/m-PVOH/PLA + 0%wax films, respectively. A drop in the films’ transparency was observed by incorporating the wax in the PLA coating layer, the extent of which was more significant with increasing wax concentration.

The films exhibited significant potential in shielding UV- light, as highlighted by the UVA- and UVB-blocking % values reported in Table 5, which are equal or higher than 92.0% and 99.2% for all the samples, respectively. The UV-light screening ability of these films plays a fundamental role in preserving the chemical, physical, and biological properties of food products, since UV radiation primarily induces light-induced oxidation in protein-rich and high-fat foods [69].

## 4. Conclusions

This study aimed to investigate the transport properties of high-barrier biodegradable films made by the deposition of a double coating layer of m-PVOH and PLA + EBS wax on a PBAT/PLA (Biofilm) substrate, exploring the effect of relative humidity on barrier performance and the suitability of the structures for MAP applications. The deposition of the first coating of m-PVOH on the Biofilm web layer resulted, in the range of 30–50% RH, in a one-order-of-magnitude decrease in the water vapor permeability compared to the pure substrate, from ~67.0 to 3.5 g mm/ m^2^ d bar for the Biofilm and Biofilm/m-PVOH samples, respectively. The water vapor barrier properties remained constant up to 60% RH, above which the plasticization of the m-PVOH chains prevailed, entailing a faster diffusion. This result is of particular significance with respect to the unmodified PVOH grades, which showed no water vapor resistance and a fast increase in the P_WV_ already at RH ≥ 30%. 

The films’ barrier performances were not considerably affected by the addition of the second PLA layer up to 85% RH. Nonetheless, the incorporation of the wax in the PLA coating was effective in slowing down the permeation phenomenon by hindering the permeation of water vapor molecules and partially inhibiting the swelling of the sensitive PVOH matrix. The maximum decrease in P_WV_ with respect to the Biofilm/m-PVOH/PLA film occurred for the Biofilm/m-PVOH/PLA + 5%wax sample and was equal to 37% at 70% RH. Further incorporation of higher percentages of EBS wax into the PLA layer did not significantly alter the water vapor barrier performance of the double-coated films.

The trends of P_WV_ evolution by increasing RH were also fitted using an exponential mathematical model. The numerical results obtained agreed with the empirical data, highlighting the efficacy of the model in predicting the water vapor permeability of the multilayer films over a wide range of relative humidities, and its utility for the characterization, designing, and optimization of their barrier performance.

For what concerns PO_2_, a dramatic increase was observed at 85% RH compared to 50% RH. Also, in this case, the wax incorporation in the PLA coating was effective in decreasing the gas permeation; for the samples loaded with different wax concentrations, a PO_2_ reduction comprised between 15% and 22%, with respect to the Biofilm/m-PVOH/PLA, was observed.

The permselectivity values of the biodegradable, double-coated films were found to be comparable to those of conventional, fossil-based polymeric films used for the MAP storage of several categories of foods, from fresh-cut agricultural products to cheese. 

These results, coupled with those of the MAP tests, endorsed the potential of the films to be used as sustainable alternatives for the preservation of foods stored under modified atmosphere packaging, with the possibility to tailor the optimal film layout on the basis of the target food’s shelf-life.

## Figures and Tables

**Table 1:** List of film samples and thickness of coating layers.

Sample Film	Wax Concentration [% w/w_PLA_]	Thickness of the m-PVOH Coating [μm]	Thickness of the PLA-Based Coatings [μm]	Total Thickness [μm]
Biofilm	0	-	-	22 ± 2
Biofilm/m-PVOH	0	10 ± 1	-	32 ± 2
Biofilm/m-PVOH/PLA	0	10 ± 1	6 ± 2	38 ± 4
Biofilm/m-PVOH/PLA + 5%wax	5	10 ± 1	6 ± 1	38 ± 5
Biofilm/m-PVOH/PLA + 10%wax	10	10 ± 1	7 ± 1	39 ± 4
Biofilm/m-PVOH/PLA + 20%wax	20	10 ± 1	8 ± 1	40 ± 3

**Table 2:** P_WV_ at 30%, 50%, 70%, 85%, and 90% RH for all the biodegradable films and percentage decrease in P_WV_ (ΔP_WV_%) for the double-coated films with wax with respect to the Biofilm/m-PVOH/PLA film, at the different relative humidities.

Sample Film	P_WV_ [g mm/ m^2^ d bar]					ΔP_WV_%		
	30% RH	50% RH	70% RH	85% RH	90% RH	70% RH	85% RH	90% RH
Biofilm	64.0 ± 2.1	67.9 ± 2.9	75.0 ± 3.0	92.0 ± 1.8	124.0 ± 6.0	-	-	-
Biofilm/m-PVOH	3.4 ± 0.2	3.5 ± 0.5	10.3 ± 1.8	52.8 ± 0.7	107.3 ± 7.8	-	-	-
Biofilm/m-PVOH/PLA	6.0 ± 0.2	5.7 ± 0.4	15.1 ± 1.1	54.0 ± 1.9	82.2 ± 4.2	-	-	-
Biofilm/m-PVOH/PLA + 5%wax	4.5 ± 0.8	4.3 ± 0.1	9.5 ± 0.6	37.0 ± 2.1	69.6 ± 0.7	37.1	31.5	15.3
Biofilm/m-PVOH/PLA + 10%wax	4.7 ± 0.7	4.9 ± 0.5	9.9 ± 0.6	37.1 ± 0.9	71.8 ± 0.9	34.4	31.3	12.7
Biofilm/m-PVOH/PLA + 20%wax	4.5 ± 0.6	4.6± 0.3	10.9 ± 0.7	35.8 ± 2.9	70.3 ± 4.5	27.8	33.7	14.5

**Table 3:** Model parameters (a, b, and c) and coefficients of determination (R^2^) for all the biodegradable films.

Sample Film	a × 10^−3^	b	c
Biofilm	5.035	10.133	65.948
Biofilm/m-PVOH	2.134	11.813	2.187
Biofilm/m-PVOH/PLA	15.727	9.409	4.750
Biofilm/m-PVOH/PLA + 5%wax	1.108	12.153	4.036
Biofilm/m-PVOH/PLA + 10%wax	20.715	14.075	4.978
Biofilm/m-PVOH/PLA + 20%wax	896.146	15.006	5.190

**Table 4:** CO_2_ permeability and permselectivity values of all the biodegradable films.

Sample Film	$P_{CO_{2}}$ [cm^3^ mm/m^2^ day bar]	Permselectivity P_CO2_/P_O2_
Biofilm	305 ± 79	8.0
Biofilm/m-PVOH	0.37 ± 0.11	3.8
Biofilm/m-PVOH/PLA	0.46 ± 0.14	2.1
Biofilm/m-PVOH/PLA + 5%wax	0.44 ± 0.30	3.7
Biofilm/m-PVOH/PLA + 10%wax	0.45 ± 0.18	4.9
Biofilm/m-PVOH/PLA + 20%wax	0.46 ± 0.10	4.3

**Table 5:** Percent transparency values and UVA- and UVB-blocking % values of all the biodegradable films.

Sample Film	T_R_ [%]	UVA-Blocking %	UVB-Blocking %
Biofilm	9.6	96.7	99.7
Biofilm/m-PVOH	17.0	92.0	99.2
Biofilm/m-PVOH/PLA	17.1	92.0	99.2
Biofilm/m-PVOH/PLA + 5%wax	14.3	93.3	99.3
Biofilm/m-PVOH/PLA + 10%wax	12.2	94.6	99.5
Biofilm/m-PVOH/PLA + 20%wax	9.1	96.6	99.7

## Data Availability

The data presented in this study are available on request from the corresponding author.
